# Diagnostic status and epidemiological characteristics of community-acquired bacterial meningitis in children from 2019 to 2020: a multicenter retrospective study

**DOI:** 10.1186/s12887-023-04469-1

**Published:** 2024-01-04

**Authors:** Juan-Juan Liu, Zhi-Wei Xu, Hui-Qing Xu, Jia-Jun Zhu, Jie-Ning Zhang, Sheng Fang, Sheng-Fu Yuan, He-Jia Ge, Hai-Jing Li, Wen-Ji Lou, Li-Hua Chen, Feng Gao, Ying-Hu Chen

**Affiliations:** 1 Department of Infectious Diseases, National Clinical Research Center for Child Health, Children’s Hospital, Zhejiang University School of Medicine, Hangzhou, 310051 Zhejiang China; 2 Department of Pediatric Infectious Disease, The Second Affiliated Hospital & Yuying Children’s Hospital of Wenzhou Medical University, Wenzhou, 325027 Zhejiang China; 3 Department of Pediatrics, Ningbo Women and Children’s Hospital, Ningbo, 315012 Zhejiang China; 4 Department of Neonatology, Women’s Hospital, Zhejiang University School of Medicine, Hangzhou, 310006 Zhejiang China; 5 Department of Pediatrics, Jiaxing Maternity and Child Health Care Hospital, Jiaxing, 314000 China; 6 Taizhou Hospital of Zhejiang Province, Taizhou, 317000 Zhejiang China; 7 Department of Pediatrics, Yuyao People’s Hospital, Yuyao, 315400 Zhejiang China; 8 Department of Pediatrics, The Second Hospital of Jiaxing, Jiaxing, 314000 Zhejiang China; 9 Department of Neonatal Intensive Care Unit, Shaoxing Maternity and Child Health Care Hospital, Shaoxing, 312000 Zhejiang China; 10 Department of Pediatrics, Jinhua Municipal Central Hospital, Jinhua Hospital of Zhejiang University, Jinhua, 321000 China; 11 Department of Neonatology, National Clinical Research Center for Child Health, Children’s Hospital, Zhejiang University School of Medicine, Hangzhou, 310051 Zhejiang China; 12 Department of neurology, National Clinical Research Center for Child Health, Children’s Hospital, Zhejiang University School of Medicine, Hangzhou, 310051 Zhejiang China

**Keywords:** Bacterial Meningitis (BM), Children, Incidence, Mortality, Pathogen, Neuroimaging

## Abstract

Community-acquired bacterial meningitis (CABM) is the main cause of morbidity and mortality in children. The epidemiology of CABM is regional and highly dynamic. To clarify the diagnostic status and epidemiological characteristics of children with CABM in this region, and pay attention to the disease burden, so as to provide evidence for the prevention and treatment of CABM. By retrospective case analysis, the clinical data of 918 CABM cases in children aged 0–14 years in Zhejiang Province from January, 2019 to December, 2020 were collected. The etiological diagnosis rate of CABM in children was 23.1%, the annual incidence rate 4.42–6.15/100,000, the annual mortality rate 0.06–0.09/100,000,the cure and improvement rate 94.4%, and the case fatality rate 1.4%. The total incidence of neuroimaging abnormalities was 20.6%. The median length of stay for CABM children was 20(16) days, with an average cost of 21,531(24,835) yuan. In addition, the incidence rate was decreased with age. *Escherichia coli(E.coli)* and g*roup B Streptococcus agalactiae(GBS)* were the principal pathogens in CABM infant<3 months(43.3%, 34.1%), and *Streptococcus pneumoniae(S. pneumoniae)* was the most common pathogen in children ≥ 3 months(33.9%). In conclusion, the annual incidence and mortality of CABM in children aged 0–14 years in Zhejiang Province are at intermediate and low level. The distribution of CABM incidence and pathogen spectrum are different in age; the incidence of abnormal neuroimaging is high; and the economic burden is heavy.

## Introduction

Community-acquired bacterial meningitis (CABM), an inflammation of meningitis influencing the pia, arachnoid, and subarachnoid space induced by bacteria and bacterial products, is a major cause of morbidity and mortality in children [1]. The epidemiology of CABM is regional and highly dynamic, affected by factors such as vaccines, climate, latitude, population movement, viral infections, and poverty. For example, in well-resourced areas, the incidence of meningitis is decreased to less than 0.5–1.5 per 100,000 population; while in the Sahel region of Africa, epidemic meningitis induced by *Neisseria meningitidis* and *Streptococcus pneumoniae* (*S. pneumoniae*) persists, with an incidence of 1000 per 100,000 cases [2]. In addition to geographical differences in the incidence of CABM, the prognosis also varies with the age of onset, regions, and causative agents [1, 3].

Studies have shown that the case fatality rate of CABM in children is 30%, and 50% of survivors suffer neurological complications [3]. These complications include seizures, focal neurological deficits, subdural effusions, hydrocephalus, hearing loss, cognitive impairment, and epilepsy, among others. Therefore, prompt assessment and immediate empirical treatment of CABM can reduce the likelihood of death outcomes and chronic neurological sequelae [3, 4]. Studies showed mortality rates of untreated BM approaching 100% [1]. An important basis for empirical treatment is the epidemiological characteristics of CABM in the region. At present, there is a relative lack of data from epidemiological studies of CABM in children in Zhejiang Province. Consequently, in this study, we employed a multicenter and large-sample retrospective case analysis to clarify the epidemiological characteristics of CABM children in Zhejiang Province. And this study could not only guide empirical treatment in the early clinical stage, but also provide an etiological basis for the immunization program of meningitis vaccine.

## Methods

### Study design

**Fig. 1:**
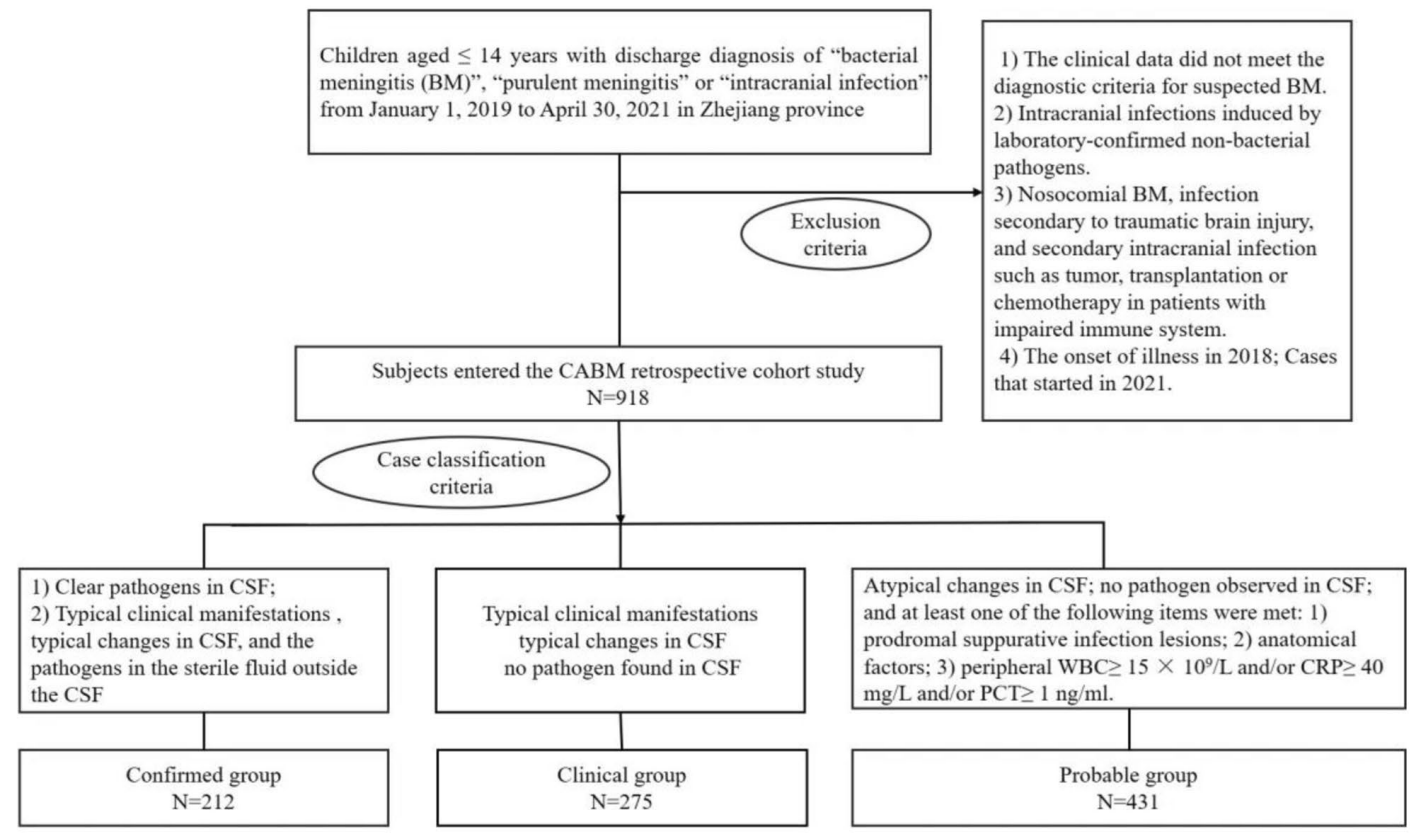
Flow diagram for the selection of subjects in the retrospective cohort study of CABM in children aged 0–14 years in Zhejiang province from 2019 to 2020 Note: 1). Some critical cases died without lumbar puncture. This type of case could be diagnosed by typical clinical manifestations and brain imaging abnormalities, such as subdural effusion/empyema, meningeal thickening, or enhanced echo of the brain ependymal. 2) The first atypical CSF change required dynamic observation of changes in indicators.

This study was a multi-center retrospective study with 50 hospitals as participators. The 50 hospitals included in the study covered almost all the hospitals with children’s inpatient departments in Zhejiang province. These hospitals were distributed in 11 prefecture-level cities in Zhejiang province, including 42 tertiary hospitals and 8 secondary hospitals. The clinical data of 918 children aged ≤ 14 years with discharge diagnosis of “bacterial meningitis (BM)”, “purulent meningitis” or “intracranial infection” from January 1, 2019 to April 30, 2021 were collected for retrospectively analysis. By referring to the electronic medical records, the clinical data of cases that met the inclusion criteria were collected, including demographics, length of hospital stay, hospitalization costs, clinical prognosis, blood and cerebrospinal fluid (CSF) sampling results (microbiologic results, CSF routine, CSF biochemistry, etc.), and head imaging results (ultrasound, CT, and magnetic resonance; all included cases completed at least one of the above imaging data). Inclusion criteria: Patients aged ≤ 14 years (including neonates) with “BM”, “purulent meningitis” or “intracranial infection” were discharged from the hospital from January 1, 2019 to April 30, 2021. Exclusion criteria: (1) Cases whose clinical data did not meet the diagnostic criteria for suspected bacterial meningitis. (2) Intracranial infections induced by laboratory-confirmed non-bacterial pathogens, including viral encephalitis, fungal encephalitis, intracranial infections with mycoplasma, rickettsia and parasitic. (3) Nosocomial BM (craniotomy, CSF leakage, intracranial catheter infection, etc.), infection secondary to traumatic brain injury, and secondary intracranial infection such as tumor, transplantation or chemotherapy in patients with impaired immune system. (4) Discharged from hospital in 2019, with the onset of illness in 2018; Cases that started in 2021. (Fig. 1)

### Case classification criteria

Grouping criteria were based on the WHO recommended case definition [5].

Pathogen confirmed (Confirmed) group: Clear pathogens were determined in CSF or blood with one of the following methods: (1) culture; (2) non-culture methods: antigen detection, Gram staining smear, nucleic acid detection. Clinical diagnosis case (Clinical) group: Typical clinical manifestations; typical changes in CSF; no pathogen found in CSF. Probable case (Probable) group: Atypical changes in CSF; no pathogen observed in CSF; and at least one of the following items were met: (1) prodromal suppurative infection lesions; (2) anatomical factors; (3) peripheral white blood cells ≥ 15 × 10^9^/L and/or high sensitive C-reactive protein ≥ 40 mg/L and/or serum procalcitonin ≥ 1 ng/ml. Typical clinical manifestations of children with clinical syndromes of suspected BM were shown as follows: fever (usually > 38.5 ° C rectal or 38.0 ° C axillary), drowsiness, confusion, severe headache, convulsions, projectile vomiting, bulging anterior fontanel, nuchal rigidity, etc. Typical changes in CSF: (A) turbid or rice soup alike appearance, and the pressure was increased (200–500 mm CSF); (B) up-regulated white blood cell count, often ≥ 1000 × 10^6^/L, and the classification was dominated by multinucleated cells (80 -90%); elevated protein concentration (> 100 mg/dl), and low CSF glucose concentration (< 40 mg/dl) [6]. Atypical changes of CSF (only 1–2 abnormalities in the following 3 items): white blood cell count of CSF was slightly high (tens to hundreds); CSF glucose was mildly low or protein was a little elevated.

### Clinical prognostic criteria

Cure: full course of anti-infection, disappearances of clinical symptoms and signs, and normal CSF indicators. Improved: full course of anti-infection, disappearances of clinical manifestations and signs, normal CSF white blood cell count; and only CSF protein and/or glucose concentration did not return to normal level. Not cured: Discharge with insufficient course of anti-infection or sufficient course but accompanied by serious complications (brain abscess, secondary epilepsy, cognitive impairment, etc.). Death.

### Statistical analysis

Spss20.0 statistical software was applied in this report. Continuous variables exhibited a skewed distribution and were expressed as M (IQR). And continuous variables between two groups and among three groups were compared through Mann Whitney U test and Kruskal-wallis test respectively. Categorical variables were displayed as number of cases n (%), and Chi-square test or Fisher’s exact test was applied to check the outcomes. *P* < 0.05 was considered as significant difference. Incidence rate = the number of new cases of a disease in a population in a certain period/the number of exposed population in the population during the same period *K. K = 100,000/100,000. Number of exposed population: people in the population of a certain area who were likely to develop the observed disease during the observation period. Therefore, individuals with the disease were not included. However, because it was difficult to divide in practical work, the average population of the area during the observation period was used as the denominator. In this study, the average population during the observation period was used as the denominator. Mortality rate = total number of deaths (due to a disease) in a period/average population in the same period ×100%. In this study, the population base of Zhejiang Province aged 0–14 years was based on the data released by the 7th national census in 2020, and the newborn population was collected from the Statistics Bureau of Zhejiang Province. The 95% confidence interval (95% CI) of the rate was calculated using the following formula: $p\pm 1.96\sqrt{\frac{pq}{n}}$, q = 1-p. The 95% CI of the rate difference was calculated as follows:

$$\left({p}_{1}-{p}_{2}\right)\pm 1.96\sqrt{\frac{{p}_{1}{q}_{1}}{{n}_{1}}+\frac{{p}_{2}{q}_{2}}{{n}_{2}} };{q}_{1}=1-{p}_{1;}\, {q}_{2}=1-{p}_{2}$$.

## Results

### Diagnostic status

A total of 918 children with CABM were included in this study, including 212 pathogen confirmed cases, 275 clinically diagnosed cases, and 431 probable cases. In this study, the etiological diagnosis rate was 23.1% (212 cases, 95% CI: 20.4, 25.8). To be specific, the positive rates of blood and CSF culture were (122 cases, 13.3%, 95% CI:11.1, 15.5) and (138 cases, 15.0%, 95%CI: 12.7, 17.3) respectively. In addition, seven pathogens were detected in CSF by metagenomic next-generation sequencing (mNGS). This study also observed that the positive rate of pathogen culture in septic shock children (55.9%, 19 cases) was much higher than that in children without shock (21.0%, 186 cases), the difference was statistically significant (*P* < 0.01).

### General clinical data

Of the 918 cases, the gender ratio between male and female was 177.9:100. Neonates accounted for the largest proportion of all cases, reaching 44.4%, and only 12.3% were 1–14 years old. The median length of hospital stay of all children was 20 (16) days, with an average cost of 21,531 (24,835) yuan. In comparisons with the clinical and probable groups, confirmed group exhibited longer length of hospital stay and higher average cost. The overall cure and improvement rate (867 cases, 94.4%, 95% CI: 93.0, 95.9) as well as case fatality rate (13 cases, 1.4%, 95% CI: 0.7, 2.2) was observed. The cure and improvement rate of confirmed group (188 cases, 88.7%) was lower than that of clinical (264 cases, 96.0%) and probable groups (415 cases, 96.3%) (Table 1).

**Table 1:** Clinical characteristics of CABM in 918 children aged 0–14 years in Zhejiang Province from 2019 to 2020

	Confirmed group N = 212	Clinical group N = 275	Probable group N = 431	All Cases N = 918	*P*
Gender n (%)					
Male	119 (56.1)	194 (70.5)	271 (62.9)	584 (63.6)	0.004
Female	93 (43.9)	81 (29.5)	160 (37.1)	334 (36.4)	
Age n (%)					
Neonates	120 (56.6)	136 (49.5)	152 (35.3)	408 (44.4)	< 0.001
1–2 months	47 (22.2)	70 (25.5)	113 (26.2)	230 (25.1)	
3–12 months	24 (11.3)	31 (11.3)	112 (26.0)	167 (18.2)	
1–14 years	21 (9.9)	38 (13.8)	54 (12.5)	113 (12.3)	
Length of stay (day)					
M (IQR)	29 (26)	23 (18)	15 (8)	20 (16)	< 0.001
Average cost CNY					
M (IQR)	35,824 (438,217)	27,768 (30,446)	16,209 (11,228)	21,531 (24,835)	< 0.001
Prognosis n (%)					
Cure and improved	188 (88.7)	264 (96.0)	415 (96.3)	867 (94.4)	< 0.001
Cure	61 (28.8)	95 (34.5)	156 (36.2)	312 (33.9)	0.017
Improved	127 (59.9)	169 (61.5)	259 (60.1)	555 (60.5)	
Not healed	18 (8.5)	8 (2.9)	12 (2.8)	38 (4.1)	
Death	6 (2.8)	3 (1.1)	4 (0.9)	13 (1.4)	

Confirmed group, Pathogenic bacteria found in cerebrospinal fluid or blood; Clinical group, Typical clinical presentation and CSF showed typical changes, but no pathogenic organism was found in the CSF; Probable group, Atypical changes in CSF and no pathogen found in CSF, but associated clinical symptoms; M(IQR), median (interquartile range); CNY, China Yuan

### Clinical distribution and outcome of confirmed cases

There were 212 children in the confirmed group. Among them, 119 cases were detected with Gram-positive bacteria (G +) and 93 with Gram-negative bacteria (G −). The onset was commonest in neonates (G + 62 cases, G − 58 cases). The cure and improvement rates of positive and negative bacteria were (108 cases, 90.8%, 95% CI: 85.5, 96.0) and (80 cases, 86%, 95% CI: 78.8, 93.2) respectively, and the case fatality rates were (1 case, 0.8%, 95% CI: -0.8, 2.5) and (5 cases, 5.4%, 95% CI: 0.7, 10.0) respectively. There was no statistically significant difference in the compositions of clinical cure, improvement, and other outcomes (including death and not healed) between the groups of G + and G- (*P* > 0.05). Besides, statistical differences between the two groups were not observed in the length of hospital stay and average cost (Table 2).

**Table 2:** Clinical characteristics of CABM in 212 confirmed cases of children in Zhejiang from 2019 to 2020

	G + group, N = 119	G- group, N = 93	*P*
Gender n (%)			
Male	60 (50.4)	59 (63.4)	0.07
Female	59 (49.6)	34 (36.6)	
Age n (%)			
Neonates	62 (52.1)	58 (62.4)	0.08
1–2 months	25 (21.0)	22(23.7)	
3–12 months	15(12.6)	9(9.7)	
1–14 years	17 (14.3)	4 (4.3)	
Length of stay (day)			
M (IQR)	29 (30)	29 (23)	0.69
Average cost CNY			
M (IQR)	39,164.7 (45,712.4)	34,626.9 (31,276.9)	0.24
Prognosis n (%)			
Cure	35 (29.4)	26 (28.0)	0.56
Improved	73 (61.3)	54 (58.1)	
Not healed	10 (8.4)	8 (8.6)	
Death	1 (0.8)	5 (5.4)	

G +: Gram-positive bacteria; G-: Gram-negative bacteria

### Age distribution of incidence and mortality

In 2019, the annual incidence of CABM in children aged 0–14 years in Zhejiang Province was 6.15/100,000 (95% CI; 5.63, 6.67), and the mortality rate was 0.06/100,000 (95% CI: 0.01, 0.11). In 2020, the incidence rate was 4.42/100,000 (95% CI: 3.98, 4.87), and the mortality rate was 0.09/100,000 (95% CI: 0.03, 0.16). The incidence of CABM in children showed a decreasing trend from 2019 to 2020, with a rate difference of 1.73/100,000 (95% CI: 1.04, 2.41). Additionally, the incidence of CABM in children was declined with age, while the morbidity was as high as 30.13–39.12 per 100,000 in the neonatal period (95% CI: 25.43, 34.82; 34.27, 43.97) (Table 3).

**Table 3:** Comparison of the incidence and mortality of CABM in children aged 0–14 years in different age groups in Zhejiang from 2019 to 2020

Age group	Incidence/100,000 (n/N)		Rate difference (95%CI)	Mortality/100,000 (n/N)		Rate difference (95%CI)
	2019	2020		2019	2020	
0–4 years	16.91 (499/2,951,489)	12.33 (364/2,951,489)	4.57 (2.62, 6.52)	0.14 (4/2,951,489)	0.27 (8/2,951,489)	-0.14 (-0.37, 0.09)
Neonates	39.12 (250/639,014)	30.13 (158/524,432)	8.99 (2.24, 15.75)	0.47 (3/639,014)	0.95 (5/524,432)	-0.48 (-1.47, 0.51)
1-2 M	18.80 (131/696,985)	16.72 (99/592,190)	2.08 (-2.53, 6.68)	0.14 (1/696,985)	0.17 (1/592,190)	-0.03 (-0.46, 0.41)
3 M-4Y	3.83 (118/3,080,217)	3.58 (107/2,991,970)	0.25 (-0.71, 1.22)	0 (0/3,080,217)	0.07 (2/2,991,970)	-0.07 (-0.16, 0.03)
5-14years	0.61 (35/5,730,292)	0.35 (20/5,730,292)	0.26 (0.01, 0.52)	0.01 (1/5,730,292)	0 (0/5,730,292)	0.02 (-0.02, 0.05)
Total cases	6.15 (534/8,681,781)	4.42 (384/8,681,781)	1.73 (1.04, 2.41)	0.06 (5/8,681,781)	0.09 (8/8,681,781)	-0.03 (-0.12, 0.05)

The published 7th census in 2020 was adopted as the population base aged 0–14 years in Zhejiang Province in this study, and the newborn birth population was obtained from Zhejiang Provincial Statistics Bureau

### Pathogen distribution and detection rate

**Fig. 2:**
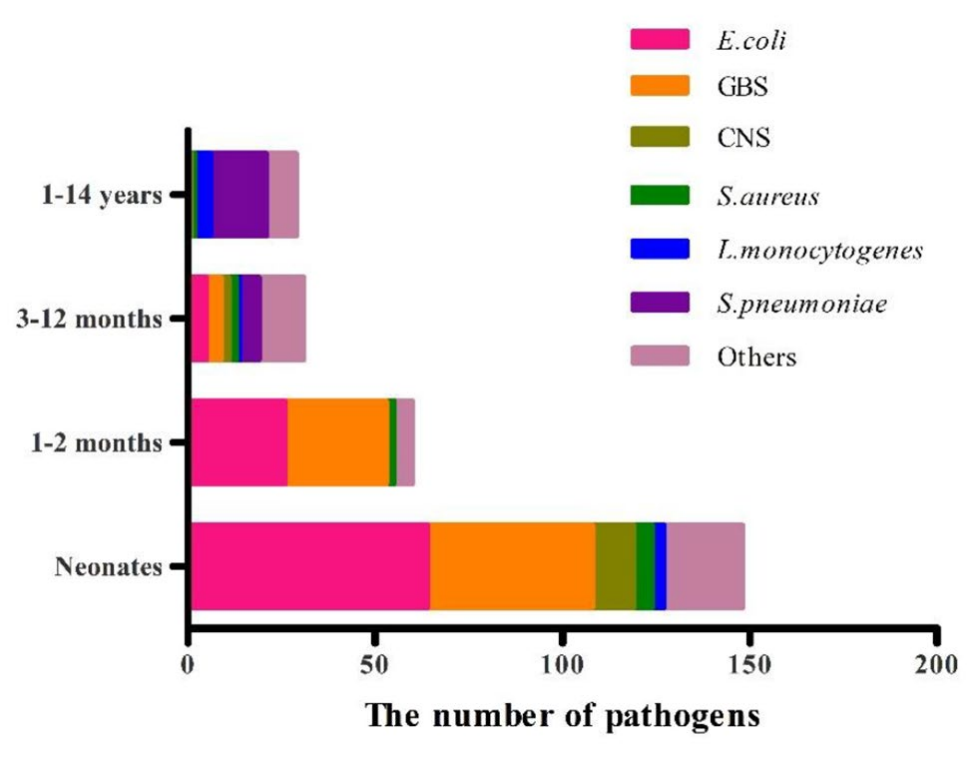
Composition of 267 pathogens detected by CABM in children aged 0–14 years in Zhejiang Province from 2019 to 2020

A total of 267 bacterial pathogens were detected from the blood and CSF of all children, including 147 G + and 120 G −. The top four pathogens of 267 strains were *E.coli*(95 cases, 35.6%), *GBS* (75 cases, 28.1%), *S. pneumoniae* (20 cases, 7.5%), and *coagulase-negative staphylococci* (*CNS*) (14 cases, 5.2%) in turn. While in newborns the top four pathogens were *E.coli* (64 cases, 43.2%), *GBS* (44 cases, 29.7%), *CNS* (11 cases, 7.4%) and *Staphylococcus aureus* (*S.aureus*) (5 cases, 3.4%) respectively. The main pathogens in children aged 1–2 months were *GBS* (27 cases, 45%) and *E.coli* (26 cases, 43.3%); aged 3–12 months were *S. pneumoniae* (5 cases, 16.6%), *E.coli* (5 cases, 16.6%), *GBS* (4 cases, 13.3%) and *Salmonella* (4 cases, 13.3%) in turn; and for children aged 1–14 years, *S. pneumoniae* (15 cases, 51.7%) was the common pathogen, followed by *Listeria monocytogenes* (4 cases, 13.8%). *E. coli* and *GBS* were the top two pathogens in infants < 3 months of age, occupying (90 cases, 43.3%) and (71 cases, 34.1%), respectively. The highest detection rate in children ≥ 3 months was *S. pneumoniae* (20 cases, 33.9%) (Fig. 2). Furthermore, the pathogen detection rate in the newborns group accounted for the highest proportion in all age groups, reaching 29.41%. And there was a statistically significant difference in the pathogen detection rate among different age groups (*P* < 0.01) (Table 4).

**Table 4:** Detection rate of pathogens in 918 children with CABM.

Age group	Organism, n (%)								
	*E.coli*	*GBS*	*S.pneumoniae*	*CNS*	*L.monocytogenes*	*S.aureus*	*Hib*	Others	All
Neonates N = 408	48 (11.76)	34 (8.33)	0 (0.00)	10 (2.45)	3 (0.74)	5 (1.23)	1 (0.25)	17 (4.17)	120 (29.41)
1–2 M N = 230	20 (8.70)	20 (8.70)	0 (0.00)	0 (0.00)	0 (0.00)	2 (0.87)	0 (0.00)	5 (2.17)	47 (20.43)
3–12 M N = 167	3 (1.80)	3 (1.80)	4 (2.40)	2 (1.20)	1 (0.60)	2 (1.20)	1 (0.60)	7 (4.19)	24 (14.37)
1–14 Y N = 113	0 (0.00)	0 (0.00)	10 (8.85)	1 (0.88)	2 (1.77)	1 (0.88)	1 (0.88)	5 (4.42)	21 (18.58)
*P*	< 0.001	< 0.001	< 0.001	0.06	0.23	0.99	0.30	0.58	< 0.001

*E.coli, Escherichia coli; GBS, Streptococcus agalactiae; S.pneumoniae, Streptococcus pneumoniae; CNS, coagulase-negative staphylococci; L. monocytogenes, Listeria monocytogenes; S. aureus, Staphylococcus aureus; Hib, Haemophilus influenzae*

### Neuroimaging findings

The incidence of abnormal head imaging in 918 children was 20.6% (189 cases, 20.6%, 95% CI: 18.0, 23.2) (Table 5). The top two changes of the incidence were subdural fluid collection/empyema and hydrocephalus, (73 cases, 8.0%, 95% CI: 6.2, 9.7), (46 cases, 5.0%, 95% CI: 3.6, 6.4), respectively. Encephalomalacia (36 cases, 3.9%), cerebral hemorrhage (16 cases, 1.7%), ependymitis (11 cases, 1.2%), cerebral infarction (9 cases, 0.98%), brain abscess (8 cases, 0.87%) and other abnormalities (33 cases, 3.6%) (such as cerebral edema, meningeal enhancement, frontal subcortical punctate abnormal signal, etc.) were also observed. The incidence of all brain imaging changes was different among the three groups. Specifically, the incidence of abnormalities was markedly higher in the confirmed and clinical groups (35.8%, 26.2%) than that in the probable group (9.5%). And the incidence of subdural fluid collection/empyema in the confirmed group (16%) was much higher than that in the clinical group (8%) and the probable group (3.9%). There was no significant difference in subdural fluid collection/empyema when gram-negative and gram-positive bacteria appeared (*P* > 0.05) (Table 6).

**Table 5:** Analysis of brain imaging results of 918 CABM children aged 0–14 years in Zhejiang Province from 2019 to 2020

Group	All imaging abnormalities n (%)	*P*	Subdural collections/Empyema n (%)	*P*	Hydrocephalus n (%)	*P*
All cases (N = 918)	189 (20.6)		73 (8.0)		46 (5.0)	
Diagnosis						
Confirmed group (N = 212)	76 (35.8)	< 0.001	34 (16)	< 0.001	22 (10.4)	< 0.001
Clinical group (N = 275)	72 (26.2)		22 (8.0)		21 (7.6)	
Probable group(N = 431)	41 (9.5)		17 (3.9)		3 (0.7)	
Age						
Neonates (N = 408)	83 (20.3)	0.37	17 (4.2)	< 0.001	21 (5.1)	0.99
1–2 months (N = 230)	40 (17.4)		24 (10.4)		11 (4.8)	
3-12months(N = 167)	38 (22.8)		25 (15.0)		8 (4.8)	
1–14 years (N = 113)	28 (24.8)		7 (6.2)		6 (5.3)	

**Table 6:** Analysis of brain imaging results of 212 pathogen confirmed CABM children aged 0–14 years in Zhejiang Province from 2019 to 2020

Group (N = 212)	All imaging abnormalities n (%)	*P*	Subdural collections/Empyema n (%)	*P*	Hydrocephalus n (%)	*P*
Organism						
G + group(N = 119)	39 (32.8)	0.29	17 (14.3)	0.43	15 (12.6)	0.23
G − group (N = 93)	37 (39.8)		17 (18.3)		7 (7.5)	
Age						
Neonates (N = 120)	36 (30)	0.25	12 (10)	0.01	9 (7.5)	0.35
1–2 months (N = 46)	18 (39.1)		9 (19.6)		6 (13.0)	
3–12 months (N = 25)	13 (52.0)		9 (36.0)		4 (16.0)	
1–14 years (N = 21)	9 (42.9)		4 (19.0)		3 (14.3)	

The age of patients with subdural fluid collection/empyema consisted of 66 cases (90.4%) under 1 year old, and 41 cases (56.2%) were < 3 months, including 17 cases of newborns (23.3%). The incidence of subdural fluid/empyema was different between age groups, and the incidence in children aged 3–12 months with confirmed pathogen was 36%. For comparisons of hydrocephalus, the incidence in the confirmed group (10.4%) and the clinical group (7.6%) were remarkably superior to that in the probable group (0.7%). There was no significant difference in the incidence of hydrocephalus in both G + and G − groups (*P* > 0.05). The age composition of children with hydrocephalus was less than 1 year of age in 40 cases (87.0%) and < 3 months in 32 cases (69.6%), including newborns in 21 cases (45.7%). The incidence of hydrocephalus varied between 4.8% and 5.3% in different age groups.

## Discussion

Meningitis is an sever infectious disease syndrome in childhood, with a huge disease burden and large regional incidence variations [4, 7]. The Global Burden of Disease study in 2016 revealed that the incidence of meningitis was as high as 207.4 per 100,000 in South Sudan and 0.5 per 100,000 in Australia [7]. Nearly 3% of deaths in children below 5 years of age worldwide are attributed to meningitis [7]. The global mortality rate of meningitis/encephalitis in children below 5 years old ranges from 21.28/100,000 to 28.1/100,000, and the case fatality rate of BM fluctuates between 3% and 7% [8, 9]. From September 2006 to December 2009, the incidence of BM in children under 5 years old ranged from 6.95/100,000 to 22.30/100,000 in four provinces of China (Shandong, Hubei, Hebei, and Guangxi) [10]. Our study revealed that the incidence of CABM was 4.42/100,000–6.15/100,000 in children aged 0–14 years and 12.33/100,000–16.91/100,000 in children under 5 years in Zhejiang Province from 2019 to 2020. And the incidence in Zhejiang Province is at the middle and low level in China.

As this study demonstrated that, the mortality rate of CABM (0.14/100,000–0.27/100,000) and case fatality rate (1.4%) in children under 5 years of age in Zhejiang Province from 2019 to 2020 were lower than in the foreign level. When it comes to the cost, the confirmed group was the highest, with an average cost of 35,824 yuan per time. The average cost and length of hospital stay of CABM in children in Zhejiang Province from 2019 to 2020 were higher than those of pneumonia in China (5,026.76 yuan; 7.63 days) [11]. And it has revealed that the disease economic burden of CABM in children is heavy.

The incidence of BM varies according to age group. In this study, the incidence of CABM in newborns in Zhejiang Province was 30.13/100,000–39.12/100,000 from 2019 to 2020, the children aged 1–2 months was 16.72/100,000–18.80/100,000, and aged 5–14 years was decreased to 0.35/100,000–0.61/100,000. Also, USA exhibited the incidence of 81/100,000 in children under two months of age from 2006 to 2007, while 0.4/100,000 in children aged 11–17 years [3]. Infants aged ≤ 2 months are high risk population with CABM, and newborns incidence is the highest. The immature immune system of infants under 2 months of age may be responsible for the above phenomenon. On the one hand, there is a lack in maternal immunoglobulin that crosses the placenta before 32 weeks of gestation, and on the other hand, phagocytic ability of neutrophils and monocytes is limited [12]. In addition, it was discovered by our study that the incidence of CABM in children in 2020 in Zhejiang Province was remarkably lower than that in 2019. And this result was considered to be correlated with the measures such as reducing population aggregation, wearing masks, quarantine and isolation after the outbreak of COVID-19. At the same time, some studies had shown that SARS-CoV2 caused a 20–30% reduction in the incidence of meningitis during the COVID-19 pandemic [2].

Due to the high mortality rate, CABM requires immediate assessment and prompt empirical therapy [4]. A total of 918 children were included in this study, involving 46.9% probable cases. The clinical manifestations or laboratory data findings were atypical in probable group and there was a great risk of missed diagnosis. However, with the case fatality rate approaching 100% for untreated BM, suspected cases need immediate actions for specific diagnosis and empirical antimicrobial therapy [1]. Importantly, early recognition and use of appropriate antibiotics are essential to minimize deaths and complications caused by BM [2]. This study enrolled the children with probable meningitis to increase clinicians’ attention to their diagnosis and treatment. The pathogenic diagnosis rate of this study was 23.1%, while the positive rate of CSF culture was only 15%. The children in this study conducted blood culture in the early stage of the disease and the CSF of a few children received mNGS detection, improving the diagnostic yield of the pathogen. Therefore, early aseptic body fluid culture is recommended for BM children in clinical practice. For another, non-culture tests should also be performed for patients who require early detection of pathogens or have received antibiotic treatment to identify the pathogen to guide treatment as soon as possible [1].

The distribution of common pathogens of CABM in children varies among age groups. A global META analysis of BM etiology manifested that *S. pneumoniae* infection was the most common cause of BM in all pediatric groups [13]. In this study, the top two pathogens in newborns and infants < 3 months in Zhejiang Province were *E.coli* (43.3%) and *GBS* (34.1%), and *S. pneumoniae* was the most common pathogen in children ≥ 3 months (33.9%). A study by Shenzhen Children’s Hospital reported that *GBS* and *E.coli* were the main pathogens in newborns, and *S. pneumoniae* was mainly observed in older children [14]. The similar results of the two studies indicated a descending trend in the incidence of *E.coli* and *GBS*, and an ascending trend in *S. pneumoniae* with the increase of the age.

In this study, subdural fluid collections/empyema and hydrocephalus were common brain imaging changes in children with CABM. The incidence of subdural fluid collections/empyema was 8% and didn’t appear obvious differences in G + and G- infections; but the incidence in the confirmed group (16%) was much higher than that in the clinical group (8%) and the probable group (3.9%). The above findings suggested that the children with positive pathogen culture at early stage suffered higher pathogen loading, more severe host inflammatory response, and were prone to subdural fluid collections/empyema, while had little correlation with the G + or G- pathogen. Compared with older children, subdural fluid collections are most commonly observed in infants (< 1 year of age) [15]. In this paper, 90.4% children with subdural fluid collections/empyema were younger than 1 year old, and the incidence of subdural fluid collections/empyema was the highest among children aged 3–12 months in the confirmed group. In addition, the incidence of hydrocephalus in CABM patients was 5%, which is significantly lower than the results of other studies (15 – 18.8%) [16, 17]. And this difference was mainly caused by the inclusion of children with probable BM in this study. The incidence of hydrocephalus in the probable group was only 0.7%, much lower than 10.4% and 7.6% in the confirmed and clinical groups. There was no significant difference in the incidence of hydrocephalus between different age groups in this study, but some study showed that hydrocephalus is more common in neonates and infants (the incidence in patients aged < 3 months reaches 14 – 27%) [17, 18]. Zhou Wei et al. revealed a higher incidence of hydrocephalus in meningitis newborns with G- [19], while the incidence was not significantly different between G + and G- in children aged 0–14 years in this study.

In this article, the overall cure and improvement rate of CABM in children in Zhejiang Province from 2019 to 2020 was 94.4%. The prognosis of the confirmed group was worse than that of the clinical group and the probable group, suggesting that the prognosis of CABM in children was related to the bacterial loading. The main experimental method to confirm the diagnosis in our study was bacterial culture and its positive rate was correlated with bacterial loading [6]. Children with negative blood culture had lower level of bacterial loading in their blood compared to infants with positive one [20]. Bacterial loading is associated with the outcome of severe infection in the host [21]. In this study, the positive rate of bacterial culture in children with septic shock was much higher than that in those without shock, indicating that children with positive bacterial culture were more likely to suffer severe infections. Besides, the overall prognosis of CABM in children in the confirmed group was not significantly different between G + and G-. This conclusion was consistent with the results of a domestic study based on the prognosis of newborns with BM [19]. In summary, the prognosis of CABM in children is linked to bacterial loading, with little correlation with the G + and G-.

### Limitations

There were some limitations in this study. First, it lacked a long follow-up, and further improvement in the evaluation and follow-up of long-term neurological complications was needed. Furthermore, the non-culture detection method of bacterium-free fluids in the early stage of the disease should be pay more attention. Finally, in calculating the annual incidence, the local population during the observation period was used as the denominator. The inclusion of individuals with preexisting conditions in the denominator may have led to an underestimation of incidence.

## Conclusions

To sum up, our study assessed the annual incidence and mortality of CABM in children aged 0–14 years in Zhejiang Province. In this study, children with CABM were classified according to their laboratory data and clinical manifestations, including pathogen confirmed group, clinical diagnosis group and probable case group. Neuroimaging abnormalities were more common in the confirmed group, and the clinical prognosis of these children was poor. In addition, our study demonstrated that infants, especially newborns, were at high risk of CABM in children. *E.coli*, *GBS* and *S. pneumoniae* are the top three pathogens of CABM in children in Zhejiang Province. Subdural fluid collections/empyema and hydrocephalus are the most common brain imaging complications. Clarifying the epidemiological characteristics of CABM in children in the region can not only guide early clinical empirical treatment, but also provide an etiological basis for the immunization program of BM.

## Data Availability

The datasets used and/or analyzed during the present study are available from the corresponding author upon reasonable request. The data cannot be made public because of privacy or ethical restrictions.

## Abbreviations and Acronyms

BM: Bacterial meningitis
CABM: Community-acquired bacterial meningitis
CSF: Cerebrospinal fluid
CT: Computerized tomography
*E.coli*: *Escherichia coli*
*GBS*: *Group B Streptococcus agalactiae*
G +: Gram-positive bacteria
G-: Gram-negative bacteria
*S. pneumoniae*: *Streptococcus pneumoniae*

## References

[1] Kim KS (2010). Acute bacterial Meningitis in infants and children. Lancet Infect Dis.

[2] Wall EC (2021). Acute bacterial Meningitis. Curr Opin Neurol.

[3] Zainel A, Mitchell H, Sadarangani M (2021). Bacterial Meningitis in children: neurological Complications, Associated Risk factors, and Prevention. Microorganisms.

[4] Beaman MH (2018). Community-acquired acute Meningitis and encephalitis: a narrative review. Med J Aust.

[5] World Health Organization (Vaccines and Biologicals). WHO-recommended standards for surveillance of selected vaccine-preventable Diseases; 2003. WHO/V&B/03. 01. WHO, Geneva.

[6] Davis LE (2018). Acute bacterial Meningitis. Continuum (Minneap Minn).

[7] GBD 2016 Meningitis Collaborators (2018). Global, regional, and national burden of Meningitis, 1990–2016: a systematic a nalysis for the global burden of Disease Study 2016. Lancet Neurol.

[8] Wright C (2021). The global burden of Meningitis in Children: challenges with Interpreting Global Health estimates. Microorganisms.

[9] Svendsen MB (2020). Neurological sequelae remain frequent after bacterial Meningitis in children. Acta Paediatr.

[10] Li Y, Acute Meningitis and Encephalitis Syndrome Study Group (2014). Population-based surveillance for bacterial Meningitis in China, September 2006-December 2009. Emerg Infect Dis.

[11] Chinese Preventive Medicine Association, Vaccine and Immunology Branch of the Chinese Preventive Medicine Association (2021). Expert consensus on immunoprophylaxis of pneumococcal Disease (2020 version). Chin J Vaccines Immun.

[12] Alamarat Z, Hasbun R (2020). Management of Acute bacterial Meningitis in children. Infect Drug Resist.

[13] Oordt-Speets AM (2018). Global etiology of bacterial Meningitis: a systematic review and meta-analysis. PLoS ONE.

[14] Shen H (2019). The etiology of acute Meningitis and encephalitis syndromes in a sentinel pediatric hospital, Shenzhen, China. BMC Infect Dis.

[15] Namani SA (2013). Early neurologic Complications and long-term sequelae of childhood bacterial Meningitis in a limited-resource country (Kosovo). Childs Nerv Syst.

[16] Olarte L (2015). Impact of the 13-Valent Pneumococcal Conjugate Vaccine on Pneumococcal Meningitis in US children. Clin Infect Dis.

[17] Hsu MH (2018). Neurological Complications in Young infants with Acute bacterial Meningitis. Front Neurol.

[18] Ouchenir L (2017). The Epidemiology, Management, and outcomes of bacterial Meningitis in infants. Pediatrics.

[19] Collaborative Study Group for Neonatal Bacterial Meningitis (2018). A multicenter epidemiological study of neonatal bacterial Meningitis in parts of South China. Zhonghua Er Ke Za Zhi.

[20] Stranieri I (2018). Assessment and comparison of bacterial load levels determined by quantitative amplifications in blood culture-positive and negative neonatal sepsis. Rev Inst Med Trop Sao Paulo.

[21] van der Poll T, Opal SM (2008). Host-pathogen interactions in sepsis. Lancet Infect Dis.

